# The Mkk2 MAPKK Regulates Cell Wall Biogenesis in Cooperation with the Cek1-Pathway in *Candida albicans*

**DOI:** 10.1371/journal.pone.0133476

**Published:** 2015-07-21

**Authors:** Elvira Román, Rebeca Alonso-Monge, Alberto Miranda, Jesús Pla

**Affiliations:** Departamento de Microbiología II, Facultad de Farmacia, Universidad Complutense de Madrid, Plaza de Ramón y Cajal s/n, E-28040 Madrid, Spain; University of Aberdeen, UNITED KINGDOM

## Abstract

The cell wall integrity pathway (CWI) plays an important role in the biogenesis of the cell wall in *Candida albicans* and other fungi. In the present work, the *C*. *albicans MKK2* gene that encodes the putative MAPKK of this pathway was deleted in different backgrounds and the phenotypes of the resultant mutants were characterised. We show here that Mkk2 mediates the phosphorylation of the Mkc1 MAPK in response to cell wall assembly interfering agents such as zymolyase or tunicamycin and also to oxidative stress. Remarkably, *mkk2* and *mkc1* mutants display related but distinguishable- cell wall associated phenotypes and differ in the pattern of MAPK phosphorylation under different stress conditions. *mkk2* and *mkc1* mutants display an altered expression of *GSC1*, *CEK1* and *CRH11* genes at different temperatures. Combined deletion of *MKK2* with *HST7* supports a cooperative role for the Cek1-mediated and CWI pathways in regulating cell wall architecture under vegetative growth. However, and in contrast to Mkc1, Mkk2 does not seem to play a role in the virulence of *C*. *albicans* in the mouse systemic model or the *Galleria mellonella* model of infection.

## Introduction

Maintenance of fungal integrity relies on proper signalling mechanisms within the cell. Potentially harmful environmental conditions trigger signalling pathways that lead to appropriate transcriptional responses. While these responses are important to every living organism, they are crucial in pathogens, where continuous challenge to host defences may lead to efficient eradication of the pathogen or lead to the development of disease. *Candida albicans* is an opportunistic pathogen that inhabits the gastrointestinal and vaginal tract of humans, being able to cause different diseases upon alteration of host defences. Within these niches, *C*. *albicans* may be exposed to changes in pH, oxidative stress, detergents or interactions with other components of the host microbiota and host immune defences that trigger in the fungus a coordinated response. In *C*. *albicans*, as in almost every eukaryotic cell, different MAPK pathways have been described to play an essential role in these processes [1]. The Cek1 MAPK was isolated as a dominant negative gene that interfered with pheromone-mediated cell cycle arrest in *Saccharomyces cerevisiae* [2]. In *C*. *albicans*, *Cek1* was later shown to be involved in invasive growth [3] and participate in cell wall construction [4]. Cek1 receives the input via the Sho1 [4], Msb2 [5] and Opy2 [6] membrane proteins which connect the triggering stimulus through Cst20 to the Ste11-Hst7-Cek1 MAPK cascade core. Cek1 also participates in the white-cell pheromone response (involved in biofilm formation) and the opaque-cell pheromone response (involved in mating). Transcription factors mediating these responses are Tec1 and Cph1 respectively [7–9]. Cek1 is a member of the formerly called SVG pathway (Sterile-Vegetative Growth) [10] that in *S*. *cerevisiae* mediates cell wall growth under vegetative conditions. The HOG (High Osmolarity Glycerol response) pathway is important for adaptation to osmotic and oxidative stress [11–14] but is also involved in morphogenesis and virulence [15, 16]. This pathway also participates in cell wall biogenesis as shown by the decreased susceptibility of *hog1* mutants to certain antifungals such as Congo Red and Calcofluor White [15], which suggests a connection of this pathway with chitin synthesis [17]. The situation is complex, as deletion of *HOG1* results in an enhanced basal activation of Cek1 while diminishes the activation of the cell integrity MAPK (Mkc1) upon different stresses, indicating the existence of cross-talk mechanisms among these routes [4, 18–20].

The cell wall integrity (CWI) pathway is mediated by the *MKC1* gene, originally cloned by its ability to complement *S*. *cerevisiae slt2* mutants thermosensitivity [21]. In *S*. *cerevisiae*, activation of Slt2 (the homologue of Mkc1) is dependent on the presence of different membrane sensors (Wsc1, Wsc2, Mid2 and Mtl1) which connect to the conserved MAPK core: Bck1-Mkk1/Mkk2–Slt2 (see [22] for a review). In this yeast, two redundant MAPKKs have been described, *ScMKK1* and *ScMKK2* [23], both being able to interact not only with Slt2 [24] but also Mkc1 [25] in a *S*. *cerevisiae* two hybrid system. Interestingly, ScMkk2 and ScMkk1 are able to be phosphorylated by Slt2 in a complex feedback mechanism which modulates the activity of Slt2 [26]. The relevance of this route in *C*. *albicans* is revealed by the fact that *mkc1* mutants are sensitive to different antifungals such as azoles, echinocandins and cell wall degrading enzymes [25]. Mkc1 is also involved in biofilm formation being activated by surface contact that presumably facilitates invasion of solid surfaces [27]. Mkc1 is activated in response to a wide variety of stresses [20] and plays a role in virulence in the mouse systemic model [28]. Its role in promoting cell integrity seems especially relevant under temperature stress [21, 29]. Mkc1 is a client protein to the Hsp90 chaperone [30], which controls antifungal resistance in close connection with the calcineurin pathway [31]. While phenotypic analyses indicate close similarities between *S*. *cerevisiae* and *C*. *albicans* CWI routes, there also seem to exist important differences. A relevant feature is the presence in *C*. *albicans* of a single MAPKK, named Mkk2. Given the potential role of this pathway in antifungal discovery, we were interested in knowing the role of this gene in the biology and pathogenesis of this fungus. We demonstrate here that it participates in fungal cell wall construction showing similar, but also distinct, phenotypes with those displayed by *mkc1* mutants.

## Materials and Methods

### Ethics statement

All the animal experiments performed in this work were carried out in strict accordance with the regulations in the ‘‘Real Decreto1201/2005, BOE 252” for the Care and Use of Laboratory Animals of the ‘‘Ministerio de la Presidencia”, Spain. The protocol was approved by the Animal Experimentation Committee of the University Complutense of Madrid (Permit Number: BIO2012-31839–1). Animals were monitored every 12 hours (approx., first 5 days) or daily (days 5 to 15) and all efforts were made to minimise suffering. When visible signs of disease where observed (reluctance to move, disorientation and/or lack of mobility), mice were immediately euthanised. The procedure used was by CO_2_ inhalation following standard protocols (AVMA Guidelines for the Euthanasia of Animals: 2013 Edition). Mice were also euthanised at the end of the experiment (15 days). The number of animals used in the experimentation was minimised for ethical reasons.

### Strains and growth conditions

Yeast strains used in this work are summarised in Table 1. Unless otherwise stated, the mutant strains used here are all homozygous null mutants (deletion of both alleles) in a heterozygous (*URA3/ura3*) background. Yeast strains were routinely grown in YPD medium (1% yeast extract, 2% peptone, 2% glucose) at 30°C. Drop susceptibility tests were performed by adjusting cell suspension to 10^8^ cells/mL; then 1/10 serial dilutions of cells were spotted on plates supplemented with the compound under analysis at the indicated concentration. Plates were incubated at 37°C (unless otherwise stated) until they were scanned. The colony morphology was tested using Spider medium (1% mannitol, 1% nutrient broth, 0.2% K_2_HPO_4_, and 1.35% agar) and incubated for seven days at 37°C as previously described [32]. We have avoided using the prefix Ca to refer to *C*. *albicans* genes, but used instead the prefix Sc to refer to the corresponding *S*. *cerevisiae* gene only where confusion could occur.

**Table 1:** *C*. *albicans* strains used in this study.

Strain	Genotype	Nomenclature in Manuscript and Figures	Source
CAF2	*URA3/ura3*Δ::*imm434*	wt	[65]
RM100	*ura3*Δ::*imm434/ura3*Δ::*imm434 his1*Δ::*hisG/his1*Δ::*hisG-URA3-hisG*		[15]
CNC13	*ura3*Δ::*imm434/ura3*Δ::*imm434 his1*Δ::*hisG/his1*Δ::*hisG hog1*Δ::*hisG/hog1*Δ::*hisG-URA3-hisG*	*hog1*	[11]
BRD3	*ura3*Δ::*imm434/ura3*Δ::*imm434 his1*Δ::*hisG/his1*Δ::*hisG pbs2*Δ::*cat/pbs2*Δ::*cat-URA3-cat*	*pbs2*	[18]
CDH10	*ura3*Δ::*imm434/ura3*Δ::*imm434 hst7*Δ::*hisG/hst7*Δ::*hisG-URA3-hisG*	*hst7*	[66]
CM-1613	*ura3*Δ::*imm434/ura3*Δ::*imm434 mkc1*Δ::*hisG/mkc1*Δ::*hisG-URA3-hisG*	*mkc1*	[21]
AMB1	*URA3/ura3*Δ::*imm434 MKK2/mkk2*Δ::*SAT1-FLIP* ^*b*^		This work
AMB2	*URA3/ura3*Δ::*imm434 MKK2/mkk2*Δ::*FRT*		This work
AMB3	*URA3/ura3*Δ::*imm434 mkk2*Δ::*FTR/mkk2*Δ:: *SAT1-FLIP* ^*b*^		This work
AMB4	*URA3/ura3*Δ::*imm434 mkk2*Δ::*FTR/mkk2*Δ::*FTR*	*mkk2*	This work
AMB5	*ura3*Δ::*imm434/ura3*Δ::*imm434 mkc1*Δ::*hisG/mkc1*Δ::*hisG-URA3-hisG MKK2/mkk2*Δ::*mkk2*Δ::*SAT1-FLIP* ^*b*^		This work
AMB6	*ura3*Δ::*imm434/ura3*Δ::*imm434 mkc1*Δ::*hisG/mkc1*Δ::*hisG-URA3-hisG MKK2/mkk2*Δ::*FRT*		This work
AMB7	*ura3*Δ::*imm434/ura3*Δ::*imm434 mkc1*Δ::*hisG/mkc1*Δ::*hisG-URA3-hisG mkk2*Δ::*FTR/ mkk2*Δ::*SAT1-FLIP* ^*b*^		This work
AMB8	*ura3*Δ::*imm434/ura3*Δ::*imm434 mkc1*Δ::*hisG/mkc1*Δ::*hisG-URA3-hisG mkk2*Δ::*FTR/mkk2*Δ::*FTR*	*mkc1 mkk2*	This work
AMB9	*ura3*Δ::*imm434/ura3*Δ::*imm434 hst7*Δ::*hisG/hst7*Δ::*hisG-URA3-hisG MKK2/ mkk2*Δ::*SAT1-FLIP* ^*b*^		This work
AMB10	*ura3*Δ::*imm434/ura3*Δ::*imm434 hst7*Δ::*hisG/hst7*Δ::*hisG-URA3-hisG MKK2/ mkk2*Δ::*FRT*		This work
AMB11	*ura3*Δ::*imm434/ura3*Δ::*imm434 hst7*::*hisG/hst7*::*hisG-URA3-hisG mkk2*Δ::*FTR/ mkk2*Δ::*SAT1-FLIP* ^*b*^		This work
AMB12	*ura3*Δ::*imm434/ura3*Δ::*imm434 hst7*::*hisG /hst7*::*hisG-URA3-hisG mkk2*Δ::*FTR/mkk2*Δ::*FTR*	*hst7 mkk2*	This work
AMB13	*ura3*Δ::*imm434/ura3*Δ::*imm434 pbs2*Δ::*cat/pbs2*Δ::*cat-URA3-cat MKK2/mkk2*Δ::*SAT1-FLIP* ^*b*^		This work
AMB14	*ura3*Δ::*imm434/ura3*Δ::*imm434 pbs2*Δ::*cat/pbs2Δ*:: *cat-URA3-cat MKK2/ mkk2*Δ::*FRT*		This work
AMB15	*ura3*Δ::*imm434/ura3*Δ::*imm434 pbs2*Δ::*cat/pbs2*Δ::*cat-URA3-cat mkk2*Δ::*FTR/mkk2*Δ::*SAT1-FLIP* ^*b*^		Thiswork
AMB16	*ura3*Δ::*imm434/ura3*Δ::*imm434 pbs2*Δ::*cat/pbs2*Δ::*cat-URA3-cat mkk2*Δ::*FTR/mkk2*Δ::*FTR*	*pbs2 mkk2*	This work
AMB17	*URA3/ura3*Δ::*imm434 mkk2*Δ::*FTR/mkk2*Δ::*FTR ARD1/ard1*Δ::*MKK2-SAT1*	*MKK2* ^*reint*^	This work

### Molecular biology procedures and plasmid constructions

For the disruption of the *MKK2* gene, the primers o-1MKK2 (GGTACCAGGAAGGGAATGTATAATGACAA) and o-2MKK2 (CTCGAGATCTCTTTTGGGAGGTCTTCTTC) were used to amplify a 712 bp fragment flanking the 5´ region of the *MKK2* ORF; similarly, o-3MKK2 (GCGGCCGCTGATGAATGGTGCTTGATCC) and o-4MKK2 (GAGCTCGCACAAATAAACAGATACACAAACG) were used to amplify a 832 bp fragment flanking the 3´region of the *MKK2* ORF. Both fragments were subcloned in the cloning vector pGEM-T (Promega) and excised as a *Kpn* I-*Xho* I and *Not* I-*Sac* I fragments respectively. These fragments were then cloned into the *C*. *albicans* disruption plasmid pSFS2a [33] which carries the nourseothricin resistance marker (*SAT1*) and the 2 μ flipase gene (*FLP*) under the control of the *MAL2* promoter flanked by FRT sequences. The plasmid generated, pDMKK2, was digested with *Kpn* I and *Sac* I for integration in the *C*. *albicans* genome. Two rounds of integration/excision were necessary to generate a homozygous deletion mutant in the CAF2 background following the method described previously [33]; noursethricin concentration for transformant selection was 200 μg/mL. Correct integration and excision were tested by Southern blot. A similar procedure was followed to delete the gene in the other strains (*mkc1*, *hst7* and *pbs2*). For *MKK2* reintegration, a fragment of 2794 bp that includes both the *MKK2* ORF and 938 pb upstream was amplified by PCR with the primers o-MKK2-R-up (TTGAACTCGAGTGTTGGTGGTAAAAGAGCTGCAGC) and o-MMK2-R-low (GGTAATCGATAACACAGATGGAATAG). This fragment was subcloned in the cloning vector pGEM-T (Promega), excised with *Hind* III-*Xho* I and inserted in the pDARD1 vector [16] digested with the same pair of enzymes. The resulting plasmid, pDMKK2-R, was digested with *Pvu* I *Sac* I for reintegration in the *ARD1* locus.

### Growth inhibition assays

To measure growth inhibition caused by either zymolyase or tunicamycin, cell cultures were inoculated from an overnight culture to an O.D. _620 nm_ = 0.025 in YPD medium supplemented with different amounts of either zymolyase100T (ICN Biomedicals, Inc., dissolved in Tris-HCl [pH 7.5]–5% glucose) or tunicamycin (Sigma-Aldrich, dissolved in DMSO). The assay was performed using duplicate rows of a 96-well plate and incubated overnight at 37°C. Graphs represented the percentage of growth estimated by O.D. _620 nm_ measures for each strain in YPD plus both compounds compared to similar untreated cells.

### Protein extracts and immunoblot analysis

Yeast strains were exposed to different compounds for different times, indicated in each case. Samples were then collected on ice and processed for immunoblot following the protocol previously described [34]. To equalise the amount of protein loaded, samples were analysed by measuring the A_280 nm_ and then by Coomassie staining and 50 μg of protein was used for western blot. Blots were probed with anti-phospho-p38 MAP kinase (Thr180/Tyr182) 28B10 monoclonal antibody for Hog1-P detection (Cell Signaling Technology); ScHog1 (y-215) polyclonal antibody for detection of Hog1 (Santa Cruz Biotechnology), anti-phospho-p44/p42 MAPK (Thr^202^/Tyr^204^) antibody (Cell SignalingTechnology, Inc.) was used for Cek1-P and Mkc1-P detection. Cek1 and Mkc1 protein levels were determined using previously described polyclonal sera [4, 20].

### Virulence assays

Female BALB/c mice obtained from Harlan Laboratories Inc. (Italy) were used within an age of 7 to 10 weeks-old and around 18 g weight. Mice housing and other non-invasive procedures were performed in the animal facility from the Medical School of the Universidad Complutense de Madrid. Virulence of *C*. *albicans* strains was tested using a murine systemic infection model described previously [28]. Briefly, mice were randomly separated into groups of 10 mice. This number was chosen using a Power and Sample Size Software (http://biostat.mc.vanderbilt.edu/PowerSampleSize) using an independent t-test assay, with the parameters α = 0.05, Power = 0.90, difference in population means = 3, within group deviation = 2, and ratio of groups = 1. Cells of strains to be tested were collected from fresh YPD plates, washed twice with PBS and recovered by low speed centrifugation; then, 10^6^ yeast cells (in 250 μL) were inoculated into the lateral tail vein of BALB/c mice and the mortality was followed during 15 days. For the infection of *Galleria mellonella* we used a standard protocol [6]. Briefly, 10^6^ cells from overnight cultures grown in YPD at 30°C and washed in PBS were inoculated (10 μL) in the hemocoel at the last pro-leg with a Hamilton syringe. Groups of 20 larvae were used for each strain with an approximate weight of 300 mg and survival was monitored during the next 4 days. This number provides a Power = 0.95 using the above mentioned Power and Sample Size Calculation software using an independent t-test assay, with the parameters α = 0.05, difference in population means = 1.2, within group deviation = 1 and ratio of groups = 1. Kaplan–Meier survival curves are shown.

### Quantitative reverse transcription-PCR assay

Overnight *C*. *albicans* cultures incubated at 24°C were diluted to an O.D. _620 nm_ = 0.1 in pre-warmed YPD at 24°C, 30°C, 37°C and 42°C and incubated for 1 h before being collected. Total RNA was isolated from cells using the RNeasy MINI kit (Qiagen, Hilden, Germany) and following the mechanical disruption protocol provided by the manufacturer. RNA concentrations were determined by measuring absorbance at 260 nm in a nanodrop spectrophotometer (ThermoScientific NanoDrop 2000C). First-strand cDNAs were synthesised from 2 μg of total RNA, using the Reverse Transcription System (Promega) following the recommendations of the manufacturer. Quantitative reverse transcription-PCR assay was performed following the protocol described previously [35] using SYBR Green Universal Master Mix (Applied Biosystems). Real-time PCR conditions were selected according to the Universal conditions (default conditions) recommended by the manufacturer of the instrument. Each cDNA was assayed in triplicate PCR reactions. The quantification of the abundance of each transcript was determined relative to the amount of the standard transcript of *ACT1* at 24°C, and the final data on relative gene expression between the two conditions tested were calculated following the 2^-ΔΔCt^ method [36]. The primers were designed using the Primer Express Software 2.0 (Applied Biosystems). Primers Tm was close to 60°C and amplicons between 74 and 121 bp. The specificity of the primers was tested previously performing a melting curve analysis. The following forward and reverse primers were used as internal control o-ACTQTup (TGGTGGTTCTATCTTGGCTTCA); o-ACTQTlw (ATCCACATTTGTTGGAAAGTAGA). To quantify the expression of *CEK1* the primers used were: o-CEK1QTup (TTAGAAATTGTTGGAGAAGGAGCAT) and o-CEK1QTlw (GCAACTTTTTGTTGTGATGGTTTATG). *GST1* and *CRH11* expression was quantified using o-GSC1QTup (TCAACAACAACCATATGACATGGA), o-GSC1QTlw (ACCGCCATAACTAAAGTCAGAAAAG), o-CRH11QTup (ATCAAGAAATTGGAAAGTGGACAAT) and o-CRH11QTlw (AAGAGGCGGATGGACTGGGAT), respectively. Two independent experiments were done and the interexperiment Mean ± standard deviation (SD) is shown.

### Flow Cytometry and fluorescence microscopy

To quantify glucan exposure yeast cells were grown overnight in YPD at 37°C. Cells were fixed with 3.7% paraformadehyde (for 15 min at 4°C). Then, cells were collected and washed twice with PBS, incubated with 1% BSA in PBS 30 min and then incubated with anti-β(1,3)-glucan mAb (Biosupplies) for 30 min at 4°C. Then, cells were washed three times and incubated with the secondary antibody anti-IgG Alexa 488 for 30 min at 4°C. Cells were washed three times before being analysed by flow cytometry. The analyses were performed with a FACS guava easyCyte (Millipore). The relative mean fluorescence intensity is represented for each strain compared to wt strain values. The graph represents the mean of three independent experiments ± SD.

Chitin staining was performed on stationary phase cells grown at 30°C of the indicated strains. Cells were obtained by low speed centrifugation, washed twice with distilled water, fixed with 1% paraformaldehyde for 30 min. and stained at room temperature for 30 minutes with Calcofluor White (Sigma) at 50 μg/mL. Mean fluorescence intensity was determined from 50 single cell images taken in a Nikon Eclipse TE2000-U fluorescence microscope using a Hamamatsu camera and ImageJ Fiji software; the ratio to wild type is given ± SD.

### Statistical analysis

One-way or two-way ANOVA followed by Dunnett’s correction for multiple comparisons was applied to evaluate differences among mutants as indicated in the figures. Data are expressed as the mean of at least three experiments ± SD. Statistics were performed using the GraphPad software. In all cases * indicates p≤0.05, ** p≤0.01, and *** p<0.001. Survival data was analysed by Kaplan-Meier Log-Rank statistics. The Holm-Sidak post hoc test in an ANOVA test was used to evaluate significant differences in gene expression in qPCR experiments.

## Results

### Mkk2 controls the activation of Mkc1 in response to oxidative stress

**Fig 1:**
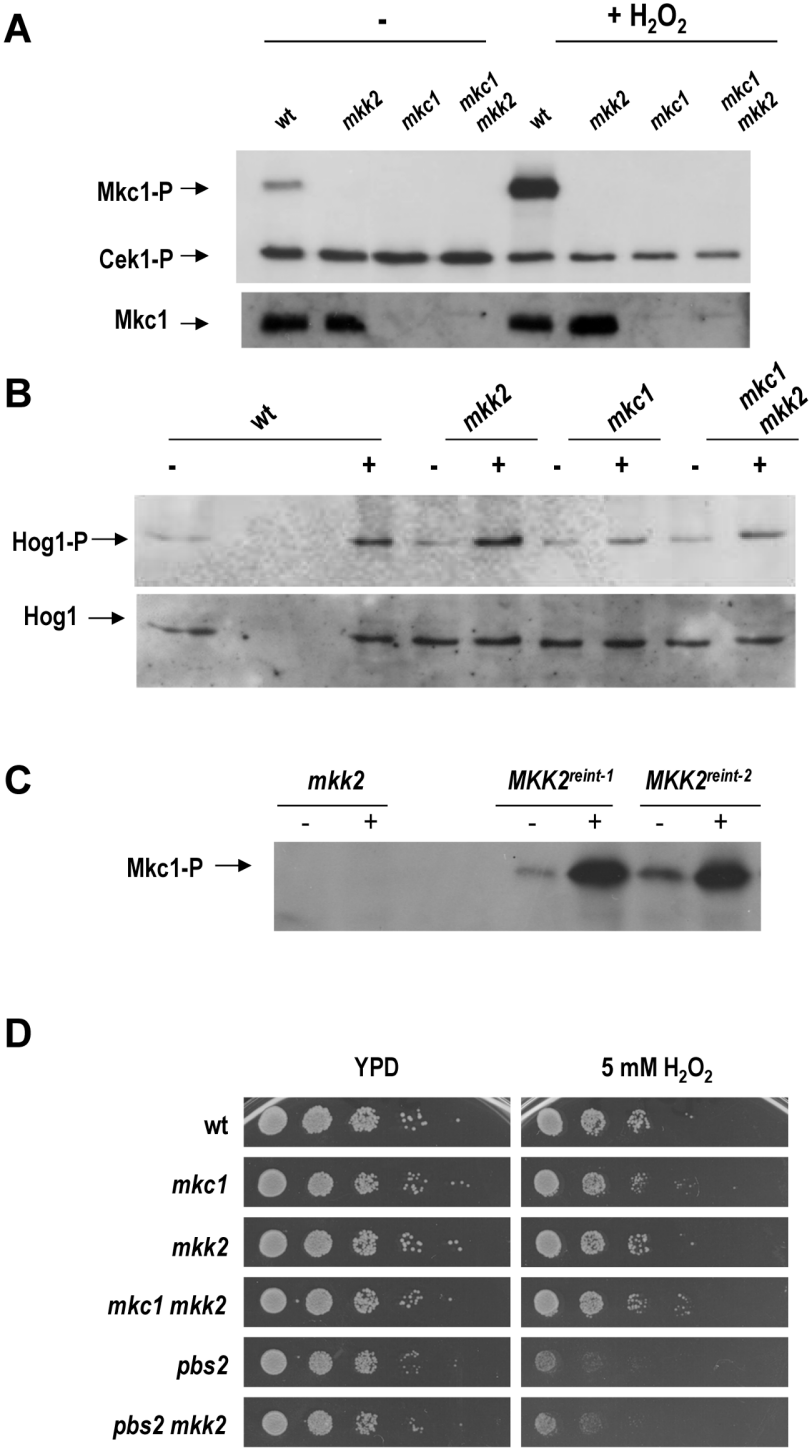
Role of CWI pathway mutants in oxidative stress. A), B) and C) Exponentially growing cells (O.D. _620 nm_ = 1) of the indicated strains were exposed (+) or not (-) to 10 mM H_2_O_2_ for 10 min and samples were processed. The phosphorylated form of the MAPKs is expressed as Mkc1-P, Cek1-P and Hog1-P. Hog1 indicates the total amount of Hog1 protein which is used as an additional loading control; Mkc1 levels were determined using a polyclonal anti-Mkc1 serum. D) Susceptibility to H_2_O_2_ was analysed by standard drop susceptibility assays. Cultures growing exponentially were plated (10 fold serial dilutions) on YPD plates supplemented or not with 5 mM hydrogen peroxide and incubated at 37°C for 24 h.

To analyse the role of the MAPKK Mkk2 in *C*. *albicans*, the gene encoding Mkk2 was disrupted using the *SAT1* flipper strategy; this system uses the dominant nourseothricin *SAT1* marker flanked by the flipase recognition site FRT [37]. *MKK2* 5’ and 3’ regions were accommodated flanking this construction and used to sequentially delete both alleles of *MKK2* (see Materials and Methods). We also deleted this gene in an *mkc1* background to carry out epistasis studies. Given the relevance of responding appropriately to oxidative stress for pathogens and the implication of Mkc1 in this kind of stress [20], we first analysed the role of Mkk2 in response to hydrogen peroxide (H_2_O_2_). Exponentially growing cultures were challenged with 10 mM H_2_O_2_ and samples were collected 10 minutes later for western blot analysis. Wild type (wt) strain displayed a basal Mkc1 phosphorylation level that increased significantly when H_2_O_2_ was added to the cultures (Fig 1A). No Mkc1 phosphorylation was detected in mutants lacking Mkc1 and/or Mkk2 neither in basal nor upon H_2_O_2_ challenge. No major changes in Mkc1 protein levels were observed in the *mkk2* background. Therefore, Mkk2 is required for Mkc1 phosphorylation upon basal and H_2_O_2_ addition. We have previously reported that the absence of Hog1 reduces the phosphorylation of Mkc1 upon H_2_O_2_ [20] or arsenate challenge [38], reflecting the crosstalk between both MAPK pathways involved in oxidative stress response. We therefore also analysed the activation of Hog1 phosphorylation in *mkk2* and *mkc1* mutants. As observed in Fig 1B, the absence of Mkk2 did not reduce the activation of Hog1 in response to H_2_O_2_. Contrary, *mkc1* cells showed reduced Hog1-P after a 10 min challenge with the oxidant. The double *mkc1 mkk2* mutant behaved closer to a single *mkc1* mutant, suggesting that this is an Mkc1-dependent phenotype.

The *MKK2* ORF under the control of its own promoter was integrated in the genome of the *mkk2* mutant in order to verify that the lack of Mkc1 activation was due to the presence of Mkk2. The generated strains were named *MKK2* ^reint-1^ and *MKK2* ^reint-2^ and included in the analysis. As expected, the reintegration of the *MKK2* gene fully allowed Mkc1 phosphorylation in response to oxidative challenge (10 mM H_2_O_2_ for 10 min) (Fig 1C) demonstrating that Mkk2 is the only MAPKK involved in Mkc1 phosphorylation upon oxidative stress response.

The susceptibility of the mutant strains was determined on YPD plates supplemented with 5 mM H_2_O_2_. As showed in Fig 1D, no significant differences were observed between wt and *mkc1*, *mkk2* and *mkc1 mkk2* mutants. Since the HOG pathway is also involved in the response to oxidative stress we wondered if the deletion of *MKK2* could influence the susceptibility of mutants in this pathway to oxidants. *MKK2* was deleted in a *pbs2* background (Pbs2 is the HOG pathway MAPKK) and susceptibility to H_2_O_2_ tested. As shown in Fig 1D, a double *pbs2 mkk2* mutant did not show an increased susceptibility to this oxidant compared to a *pbs2* strain. Collectively, these results indicate that Mkk2 plays an important role in sensing peroxide stress but it does not significantly alter the overall susceptibility to this compound in *C*. *albicans*.

### Mkk2 mediates Mkc1 phosphorylation upon treatment with cell wall disturbing compounds

**Fig 2:**
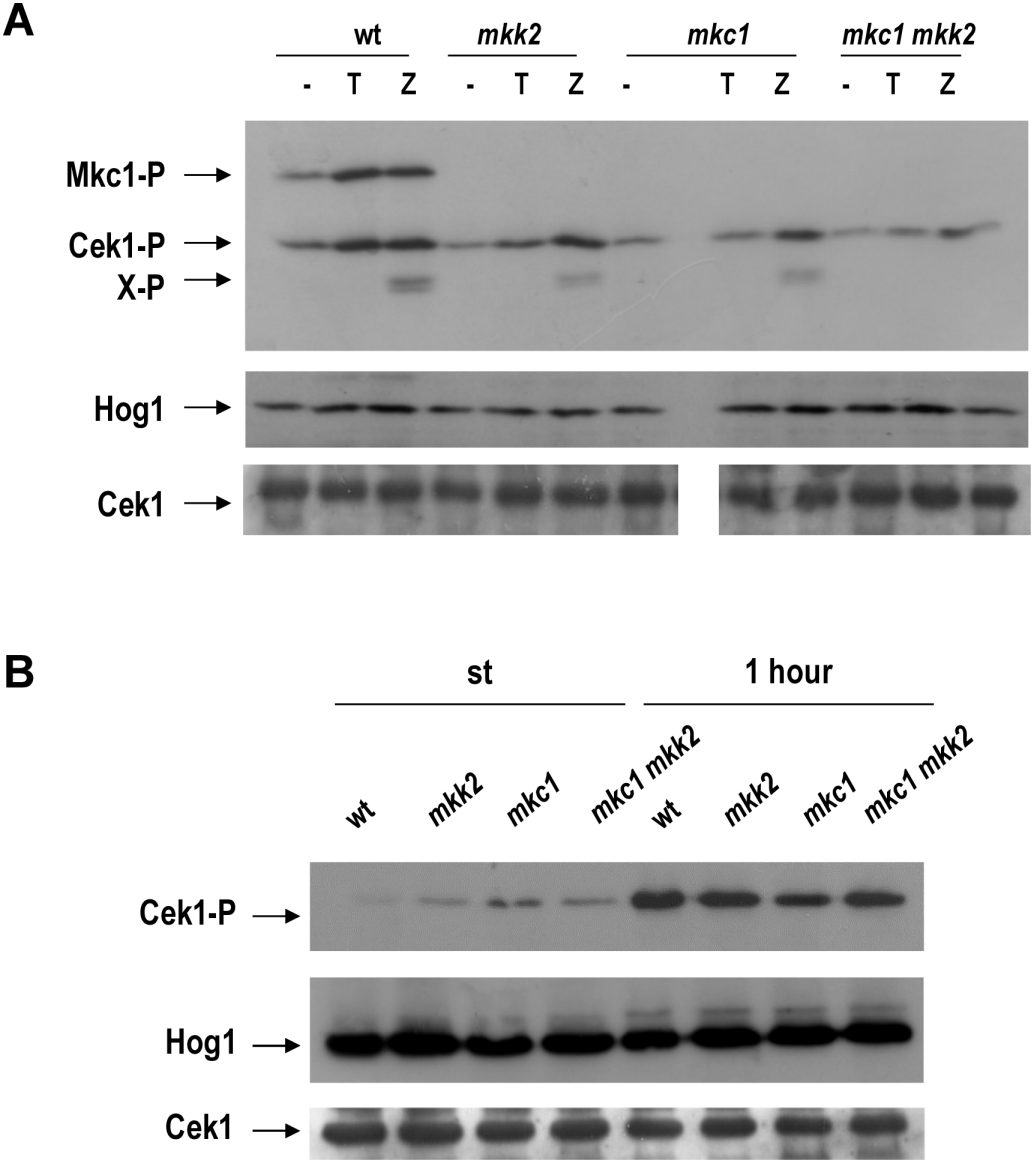
Mkk2 controls the activation of the Mkc1 MAPK upon cell wall stress. A) Effect of tunicamycin (T) and zymolyase (Z) on the pattern of Mkc1 and Cek1 phosphorylation (Mkc1-P and Cek1-P in the figure). Exponentially growing cells (O.D. _620 nm_ = 1) were challenged with 5 mg/L tunicamycin or 2 U/mL zymolyase 100-T and samples were taken after 2 hours of incubation at 37°C. X-P specifies a Cek1-P derived band. B) Cek1 activation (Cek1-P in the figure) during the resumption of growth from stationary phase. Cells were diluted in fresh YPD at O.D. _620 nm_ = 0.2 and grown at 37°C for 1 hour before collected and processed for western blot analysis.

Given the role of Mkc1 in cell wall biogenesis, the phosphorylation pattern of MAPKs was analysed upon the addition of specific drugs previously known to activate this pathway [20]. Exponentially growing cultures were challenged either with tunicamycin, an inhibitor of N-glycosylation, or with the enzymatic complex zymolyase 100-T for 2 h. Both Mkc1 and Cek1 become phosphorylated upon drug addition (Fig 2A). Mkc1 phosphorylation was completely impaired in an *mkk2* background, both under basal conditions as well as upon tunicamycin or zymolyase treatment. Also, Cek1 phosphorylation in response to tunicamycin was reduced in *mkk2*, *mkc1* and *mkc1 mkk2* backgrounds. However, the intensity of Cek1 phosphorylation was not diminished in response to zymolyase in the strains analysed. Zymolyase treatment resulted in the appearance of an additional band (named X-P in Fig 2A) which has been previously reported to be derived from Cek1-P [20]. This band was not detected in an *mkc1 mkk2* background and was less intense in the *mkk2* and *mkk1* mutant compared to the wt strain. These data indicates that the CWI pathway plays a role in Cek1 activation.

We wondered if this effect would be also detected under other activating signals. Cek1 becomes activated upon growth resumption from stationary phase cultures [4] in a process that is dependent on quorum sensing [39]. We therefore tried to determine if signals leading to this activation were dependent on *MKK2*. This was not the case and activation of Cek1 was completely independent of the presence of a functional cell integrity pathway (Fig 2B). Summarising, MAPK activation studies revealed that ^1)^ Mkk2 is the only MAPKK responsible for Mkc1 activation under our tested experimental conditions and ^2)^ there exists a crosstalk between both the Cek1 and Mkc1-mediated pathways upon cell wall disturbing compounds as evidenced by the altered Cek1 phosphorylation in CWI pathway mutants.

### *mkk2* and *mkc1* mutants displays different cell wall-related phenotypes

**Fig 3:**
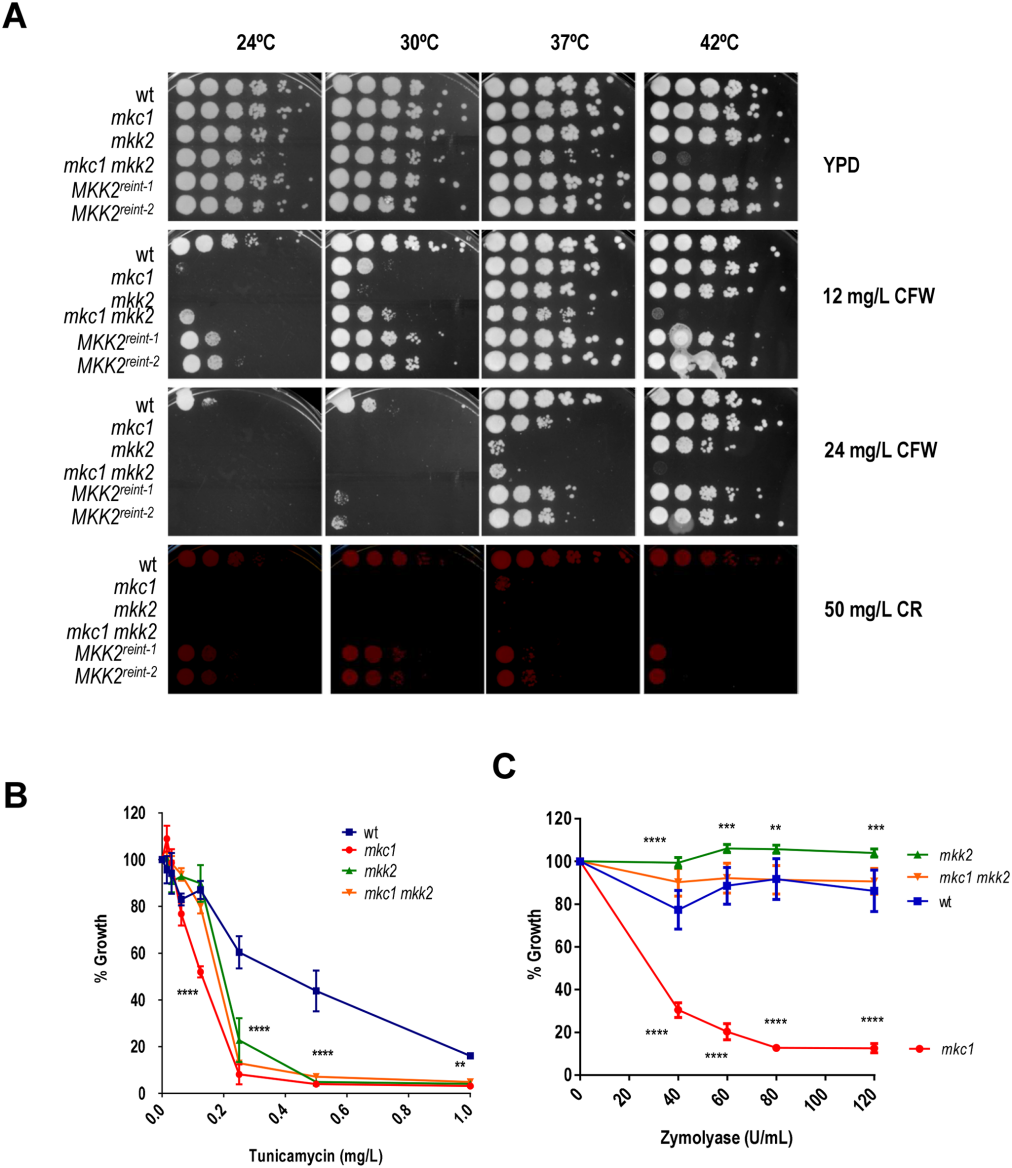
Mkc1 and Mkk2 play crucial but different roles in cell wall maintenance. A) Serial ten-fold dilutions of exponentially growing cells (O.D. _620 nm_ = 1) were spotted on YPD agar plates supplemented or not with Congo Red (CR) or Calcofluor White (CFW) and incubated at 24, 30, 37 or 42°C for 24 hours before being scanned; B and C) wt, *mkc1*, *mkk2* and *mkc1 mkk2* stationary phase cells were grown overnight at 37°C in the presence of different amounts of tunicamycin (B) or zymolyase (C) starting at O.D. _620 nm_ 0.025. Growth is depicted as the percentage of growth in YPD supplemented with cell wall interfering compounds compared to growth in YPD alone. Mean values of 3 independent experiments (two samples per experiment) are represented with bars indicating the SD (standard deviation). Two way ANOVA was used to analyse the significance of the data compare to the wild type strain (*, p<0.05; **, p<0.01, ***, p<0.001,****,p<0.0001).

In order to assess the role of Mkk2 in the biogenesis of the cell wall we analysed the susceptibility of *mkc1* and *mkk2* mutants to cell wall disturbing agents. We used Calcofluor White and Congo Red (that interfere with chitin assembly) as both are useful indicators of cell wall associated defects in yeasts. Mutants defective in both the SVG [39, 40] and CWI pathways [21] are sensitive to these drugs. These compounds were added to YPD solid plates and 10-fold dilution cell suspensions of the indicated strains growing in exponential phase were spotted. Plates were incubated at different temperatures since cell wall related phenotypes have been associated to the growth temperature. As shown in Fig 3A, *mkk2* mutants were found to be more sensitive to both compounds than the wt strain. CFW susceptibility was diminished when temperature was increased (37 and 42°C). We observed that *mkk2* and *mkc1* mutants displayed a different pattern of susceptibility to these drugs, with *mkk2* mutants being more susceptible than *mkc1*.This effect was highly dependent on the temperature (Fig 3A). The susceptibility displayed by the double *mkc1 mkk2* mutant depended on the temperature and the compounds but this double mutant was unable to grow at 42°C, even in YPD. The re-integration of *MKK2* gene improved the growth on Calcofluor White plates close to the growth of the wt strain, although they showed partial complementation, especially on Congo Red plates.

We also tested the susceptibility of *mkk2* mutants to tunicamycin in a liquid culture assay. Stationary growing cells were subjected to increasing concentrations of tunicamycin in a 96-well assay, and final growth was tested by measuring the O.D. _620 nm_ after 18 hours at 37°C. All mutant strains analysed (*mkk2*, *mkc1* and *mkc1 mkk2*) showed an increased susceptibility to tunicamycin as determined by a reduced growth compared to the wt (Fig 3B). These effects were statistically significant (shown as ***, p<0.001, two way ANOVA plus Dunnett’s multiple comparisons test) for concentrations higher than 0.2 mg/L for all strains (*mkc1*, *mkk2* and *mkc1 mkk2*) compared to wt cells. The effect of the tunicamycin depended on the temperature of growth. At 24°C *mkc1* was significantly more susceptible to tunicamycin than *mkk2*, double *mkc1 mkk2* and wild type strain. At 37°C CWI pathway mutants displayed increased sensitivity to this compound while at 42°C all the analysed strains (even the wild type) become sensitive to tunicamycin (S1 Fig). We also performed an enzymatic assay to reveal structural cell wall alterations. Cells of the indicated strains were incubated with the enzymatic complex zymolyase 100-T (an enriched β(1,3)-glucanase preparation from *Arthrobacter luteus*). Under these conditions, only *mkc1* cells were found to be sensitive to this enzymatic complex, with significant reduced growth at all activity units of zymolyase tested (Fig 3C) (shown as ***, p<0.001, two way ANOVA plus Dunnett’s multiple comparisons test). This effect was largely independent of the temperature with *mkc1* the only mutant that displayed significant susceptibility at 24, 30, 37 and 42°C (S2 Fig). Interestingly, *mkk2* mutants resisted better the presence of zymolyase compared to the wt strain (*, p<0.05, two way ANOVA plus Dunnett’s multiple comparisons test) while *mkc1 mkk2* cells showed almost a wt phenotype. Finally, the mean intensity values of Calcofluor White stained cells compared to wt cells were 1±0.26 (wt), 1.25±0.14 (*mkk2*), 1.18±0.24 (*mkc1*) and 0.980±0.26 (*mkc1 mkk2*), with no statistical significant differences, suggesting no major changes in chitin amounts. These data suggest that although both Mkk2 and Mkc1 kinases are relevant in cell wall integrity and influence cell wall architecture, they play distinct roles in cell wall biogenesis that consequently renders different phenotypes.

### Mkk2 and Mkc1 control the expression of cell wall related genes

**Fig 4:**
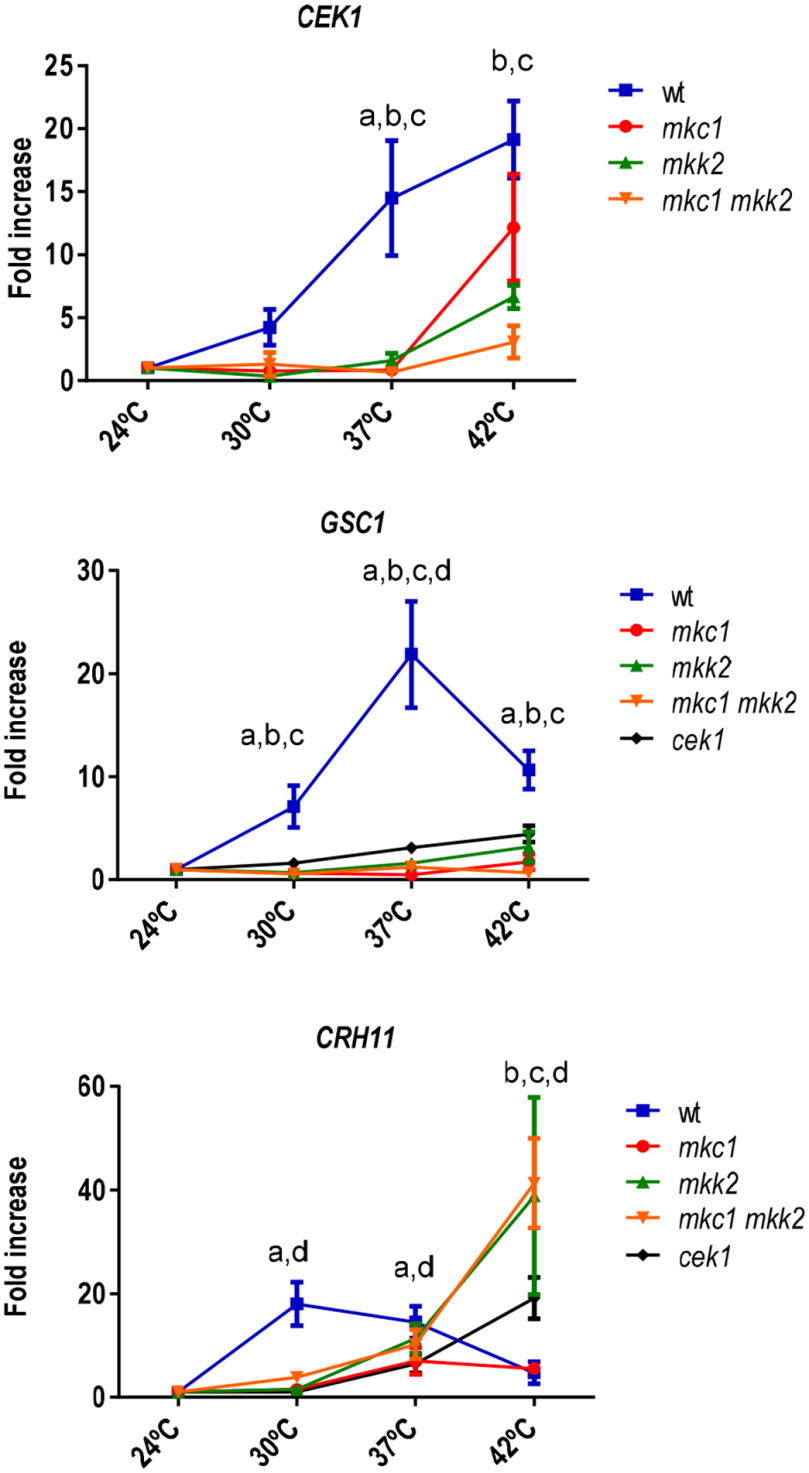
Expression of cell wall related genes in CWI mutants and *cek1* mutant. *CEK1*, *GSC1* and *CRH11* transcription levels were measured by quantitative RT-PCR. Overnight cultures at 24°C were shifted to pre-warmed YPD medium at 24, 30, 37 and 42°C and incubated for 1 h. *ACT1* mRNA was used as internal control. Therefore, values represented are the x fold increase over 24°C at each temperature. Each qPCR (2 different biological independent experiments) provided three intraexperiment values from which means were obtained (that is, intraexperimental means). Values shown in the figure represent the mean ± SD of the two intraexperimental means. The statistical significance (p < 0.05) using the Holm-Sidak post hoc test after ANOVA is shown for each value for the comparison of wt with *mkc1* (a), wt with *mkk2* (b), wt with *mkc1 mkk2* (c) and wt with *cek1* (d).

Given the phenotypic differences observed among wt, *mkc1*, *mkk2* and *mkc1 mkk2* mutants at different temperatures, we analysed the expression of different genes involved in cell wall biogenesis by qRT-PCR at 24, 30, 37 and 42°C. For this purpose, cells grown overnight at 24°C were diluted at O.D. _620 nm_ = 0.1 and allowed to grow at 24, 30, 37 or 42°C for 1 hour. The transcript of actin, encoded by *ACT1*, was used as internal control and all the values were expressed relative to *ACT1* values and 24°C values (see Materials and Methods). The selected genes for this assay were *CRH11*, *GSC1* and *CEK1*. As shown in Fig 4 (see also S1 Table), *CEK1* expression increased with temperature in wt strains (≈4x at 30°C, ≈14x at 37°C and ≈ 20x at 42°C). However, this induction was completely absent in *mkc1*, *mkk2* and double *mkc1 mkk2* mutants up to 37°C. At 42°C an increase in *CEK1* expression was detected in *mkc1* (≈12x) and to a minor extent in *mkk2* (≈6x) and *mkc1 mkk2* (≈3x) mutants. These changes are in accordance with protein levels detected by western blot (S3 Fig). This indicates that the CWI pathway positively regulates *CEK1* expression.

Another gene analysed was *GSC1* which encodes the essential β(1,3)-glucan synthase subunit [41] in *C*. *albicans*. Similarly to *CEK1*, induction of the expression of *GSC1* in the wt strain increased with the temperature, with a maximum (≈22x) at 37 but decreased at 42°C (≈10x). This induction was not observed in mutants lacking *MKC1* and/or *MKK2* at any temperature (Fig 4) indicating a complete dependence of glucan synthase on CWI pathway. The expression of *GSC1* was also dependent on the Cek1 pathway as revealed by the absence of significant increases at the different temperatures tested in a *cek1* mutant, in accordance with the already indicated dependence of *CEK1* with the CWI pathway. The third cell wall related gene tested was *CRH11*, homolog of *ScCRH1* in *S*. *cerevisiae* [42]. In a wt strain, the expression of *CRH11* increased at 30 (≈18x) and 37°C (≈15x) and at 42°C returned to values closer to 24°C. CWI pathway mutants were able to increase *CRH11* expression at 37°C although the expression levels were slightly lower than in the wt strain (≈11x). At 42°C, the *mkc1* mutant showed a *CRH11* expression decrease while *mkk2* and *mkc1 mkk2* mutants increased drastically *CRH11* expression (≈40x). In a *cek1* mutant, these values were 6x at 37°C and 19x at 42°C. These results indicate that both CWI and SVG pathways mediate the expression of *CEK1*, *GSC1* and *CRH11* and that this role is dependent on temperature of growth. The different expression pattern displayed by the different CWI pathway mutants may explain the diverse cell wall related phenotypes described above.

### Deletion of *MKK2* in a *hst7* mutant increases the susceptibility to cell wall disturbing compounds and prevents MAPKs phosphorylation

**Fig 5:**
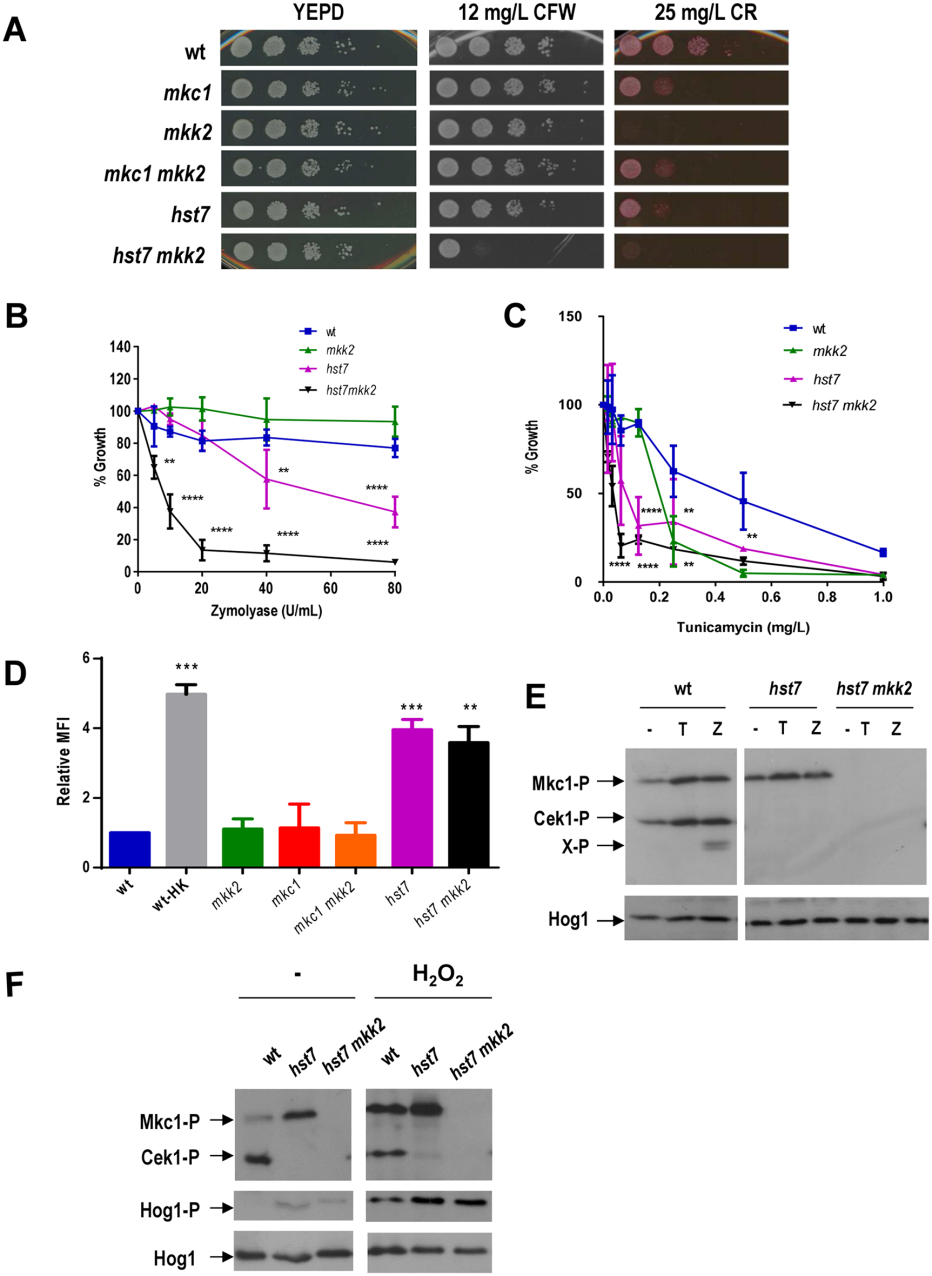
SVG and CWI pathways cooperate in cell wall biogenesis. A) 10-fold dilutions from exponentially growing cells (O.D. _620 nm_ = 1) of the indicated strains were spotted onto YPD agar plates supplemented with Congo Red (CR) or Calcofluor White (CFW) and incubated at 37°C before scanned. B) Stationary growing cells of the indicated strains were analysed for growth inhibition in the presence of zymolyase as indicated in Fig 2C (**, p<0.01; ****, p<0.0001 according to a two-way ANOVA test). C) Cultures in stationary phase of growth were refreshed in YPD medium supplemented with tunicamycin to different concentrations and incubated at 37°C overnight. Statistical differences compared to the wild type strain are indicated (**, p<0.01; ****, p<0.0001 according to a two-way ANOVA test). D) Representative flow cytometry analysis of β(1,3)-glucan exposure on live stationary *C*. *albicans* strains at 37°C. Statistical analysis of these data is shown and represents the relative mean fluorescence intensity (MFI) compared to the wt strain ± SD. Ten thousand cells were analysed in each flow cytometry run, and MFI mean values were obtained from three different experiments. One way ANOVA was used to analyse the significance of the data (**, p<0.01; ***, p<0.001). E) The effect of 5 mg/L tunicamycin or 2 U/mL zymolyase 100-T on Cek1 and Mkc1 (Cek1-P and Mkc1-P respectively) activation was tested on exponentially growing cells of the indicated strains after 2 hours of treatment at 37°C. X-P indicates a Cek1-P derived band observed upon zymolyase addition. F) Hydrogen peroxide effect on MAPK phosphorylation was analysed 10 minutes after the addition (+) or not (-) of 10 mM H_2_O_2_ (Hog1-P means phosphorylated form of Hog1).

Since our results suggest a crosstalk between CWI and SVG pathways, the *MKK2* gene was deleted in an *hst7* background, defective in the MAPKK of the Cek1-mediated pathway. *hst7* mutants show increased susceptibility to Congo Red [19]. Deletion of *MKK2* in an *hst7* background clearly aggravated the susceptibility to Calcofluor White of *hst7* cells on solid media (Fig 5A) and this effect was more evident in Calcofluor White plates supplemented with as low as 12 mg/L drug concentration. We also observed an increased sensibility to zymolyase in liquid medium (Fig 5B) in *hst7* cells (**p <0.01 at 40 U/mL and ***p<0.001 at 80 U/mL). The *hst7 mkk2* double mutant was more susceptible to zymolyase compared to single *mkk2* and *hst7* mutants and to the wt (Fig 4B), being statistically significant (***p<0.001) for all the concentrations higher than 5 U/mL. *hst7* mutants also displayed an enhanced susceptibility to tunicamycin (Fig 4C). This sensitivity was detected at lower concentration compared to *mkk2* mutants. Double *hst7 mkk2* mutants showed an aggravated phenotype at lower tunicamycin concentrations (Fig 4C).

Given the implication of β(1,3)-glucan synthase as a target of the CWI pathway, we also checked the exposure of β(1,3)-glucan in different mutants. This polymer is normally hidden within the mannoprotein layer but is easily detected upon environmental and morphological changes [43]. In *S*. *cerevisiae* and *C*. *albicans*, specific mutations have been shown to affect recognition of β(1,3)-glucan via an altered cell wall [44, 45]. As shown in Fig 5D, neither *mkc1*, *mkk2* nor *mkc1 mkk2* cells showed an altered β(1,3)-glucan exposure compared to the wt. In contrast, *hst7* cells were found to have increased (≈4x) levels and pattern of localization similar to *cek1* cells [46]. Consistent with the above results, *hst7 mkk2* mutant displayed β(1,3)-glucan exposure levels similar to *hst7* single mutant (≈3.9x). These results indicate that although *MKK2* regulates β(1,3)-glucan synthase expression, neither *MKK2* nor *MKC1* significantly alters β(1,3)—glucan exposure in the fungal surface which is mainly dependent on the Cek1-mediated pathway.

We also analysed the MAPK phosphorylation pattern by immunoblot under activating stimuli such as zymolyase and tunicamycin. First, as shown in Fig 5E, *hst7* mutants displayed an elevated Mkc1 phosphorylation under basal conditions (exponentially growing cells) and addition of cell wall impairing compounds (zymolyase or tunicamycin) led to a less pronounced increase in Mkc1 phosphorylation. No activation of Cek1 was observed in *hst7* mutants indicating that Hst7 is the only MAPK leading to Cek1 phosphorylation. As expected, *hst7 mkk2* mutants showed a complete absence of Mkc1 and Cek1 phosphorylation that correlates with the increased susceptibility to compounds that impair the cell wall architecture. In addition, the MAPK phosphorylation pattern upon H_2_O_2_ was also analysed to detect a potential cross talk among these pathways. Addition of H_2_O_2_ still enabled activation of Mkc1 in *hst7* mutants (Fig 5F), indicating that both pathways work independently under these conditions.

### Role of Mkk2 in morphogenesis and virulence

**Fig 6:**
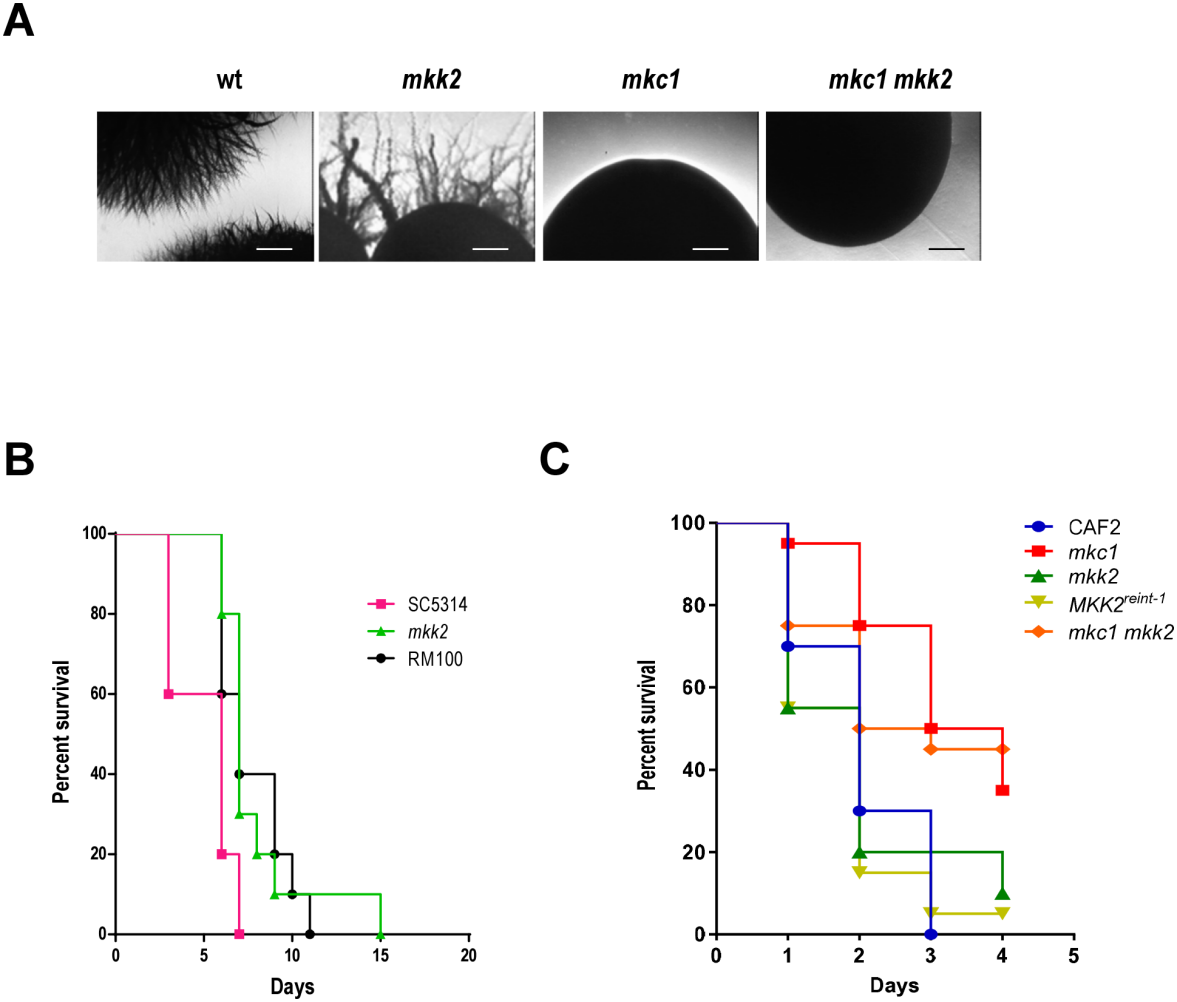
Role of Mkk2 in morphogenesis and virulence. A) Stationary phase cultures of the indicated strains were collected, washed with PBS and counted. Approximately 50 CFU (Colony Forming Units) were spread on Spider agar plates and incubated for 7 days at 37°C before colony borders were photographed. Scale bar = 200 μm. B) Survival curves of BALB/c (n = 10 in each group) infected systemically with 10^6^ cells of the *C*. *albicans* indicated strains. C) Survival curves of *Galleria mellonella* larvae (n = 20) inoculated with 10^6^ *C*. *albicans* cells is represented. Kaplan-Meier analyses were performed in both cases.

The role of *mkk2* mutants in morphogenesis was analysed on special solid media. *mkk2* cells is able to invade on Spider agar plates similarly to wt cells although invasive borders were thicker. (Fig 6A). However, *mkc1* did not display invasive borders on this solid medium and *mkc1 mkk2* double mutants behaved as *mkc1*, showing smooth colony borders (Fig 6A). Regarding filamentation in response to serum in liquid medium, CWI pathway mutants were able to form true filaments under a range of serum proportions in YPD medium similarly to wt cells (not shown). These observations indicate that Mkk2 and Mkc1 play different roles in morphogenesis.

We also analysed the relevance of a *mkk2* mutant in virulence in the standard systemic murine infection model. For this purpose, 10^6^ *C*. *albicans* cells were inoculated in the lateral tail vein of BALB/c mice. Survival of the mice was followed for 15 days and represented in a Kaplan-Meier curve (Fig 6B). *mkk2* mutants displayed a virulence similar to the wt strain RM100 (p = 0.88 using a Log-rank (Mantel-Cox) test). Median survival times were 6 days (SC5314) and 7 (*mkk2* and RM100). Differences were found between SC5314 and *mkk2* (p = 0.0030 using a Log-rank (Mantel-Cox) test) and between SC5314 and RM100 ((p = 0.008 using a Log-rank (Mantel-Cox)), reflecting most probably *URA3* genetic effects and not *MKK2* related defects, as *URA3* profoundly influences virulence in *C*. *albicans* [47, 48]. In order to confirm this result (that is, no role of *MKK2* in virulence) and compare all strains, we additionally used the *Galleria mellonella* model [49] which has been shown to correlate, to a certain extent, with the mouse systemic model [50, 51]. Under our conditions, no significant differences in the median survival time were observed in *mkk2* mutants and *MKK2* ^*reint-1*^ compared to wt cells (2 days for both strains; p = 0.8). In contrast, *mkc1* cells showed a median survival time of 3.5 days (p<0.001), in close agreement with previous observations in the mouse systemic model which indicates reduced virulence for this mutant [28]. The double *mkc1 mkk2* showed in this model an intermediate behaviour (median survival time 2.5 days) (p = 0.012). Therefore, in contrast to Mkc1, Mkk2 does not play a significant role in *C*. *albicans* virulence in two different experimental models.

## Discussion

**Fig 7:**
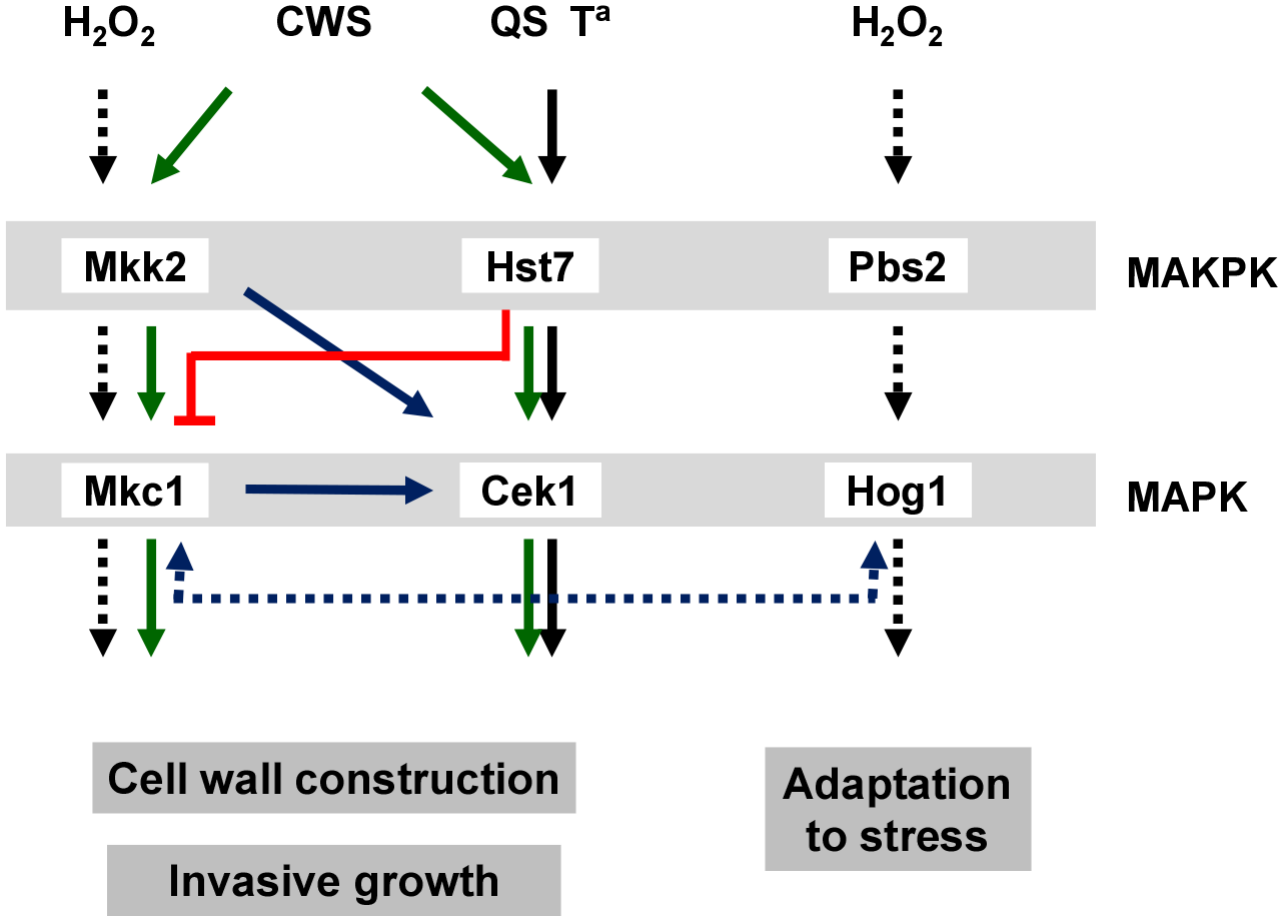
Diagram showing the main crosstalk mechanisms among the three MAPK pathways under study. Oxidative stress (H_2_O_2_, dotted line) triggers Mkc1 and Hog1 phosphorylation. Hog1 phosphorylation is relevant for cells to adapt to stress and the absence of elements belonging to this pathway prevent Mkc1 phosphorylation upon H_2_O_2_ challenge. Similarly, *mkc1* mutants reduce Hog1 phosphorylation under this stress (blue dotted line). Cell wall disturbing compounds (Cell Wall Stress, CWS) trigger Mkc1 and Cek1 phosphorylation allowing cell wall remodeling (green line). Mkk2 and Mkc1 play a positive role in Cek1 activation (continuous blue line). However, Hst7 represses Mkc1 phosphorylation upon standard growth conditions (red line). Resumption of growth from stationary phase (QS), as well as temperature increase, trigger Cek1 phosphorylation and induce the expression of Cek1, Chr11 and Gsc1 in a Mkk2 and Mkc1-dependent manner.

The current work reports the role that the Mkk2 MAPKK plays in the opportunistic pathogen *C*. *albicans*. Unlike *S*. *cerevisiae* that has two MAPKK proteins in the CWI pathway, ScMkk1 and ScMkk2, only one gene named *MKK2* is present in *C*. *albicans*. Mkk2 shares an overall 50% identity (66% similarity) to both ScMkk1 and ScMkk2; this similarity extends from amino acid 147 to the C-terminus of the protein, while there is substantial divergence in the first (1–146) N-terminal portion of the protein. Curiously, ScMkk1 and ScMkk2 N-terminal domains were reported to play an important role in the interaction with the Slt2 MAPK [24] and Mkc1 [25]. Although we have not tested the physical interaction between Mkk2 and Mkc1, the genetic relationship was evident since no Mkc1 phosphorylation was detected in the absence of Mkk2 (Fig 1A). This data supports the epistatic relationship between Mkk2 and Mkc1 and the existence of a unique MAPKK in the *C*. *albicans* CWI pathway responsible for the transmission of both cell wall and oxidative stress signals (Fig 7). Oxidative stress (through Reactive Oxygen Species (ROS)) also activates Hog1 [14] via the Ssk1 [52] and Ssk2 [53] proteins. *hog1* mutants fail to efficiently activate Mkc1 [18] and, conversely, *mkc1* mutants show defects in Hog1 activation [20], suggesting the existence of a mechanism of control that prevents activation of only one of these MAPK pathways under oxidative stress. However, while Hog1 activation results in glycerol accumulation [11] and resistance to stress [12, 14], the physiological role of Mkc1 activation via ROS is currently unknown. In addition, the precise point where the signal generated by ROS feeds into the CWI MAPK pathway is not known, although this work indicates that it occurs upstream Mkk2. We show here that the MAPKK Mkk2 performs an important function in the construction of the cell wall. *mkk2* mutants were found to be sensitive to cell wall interfering drugs such as tunicamycin, Congo Red and Calcofluor White compared to wt cells. Although *mkc1* and *mkk2* mutants displayed cell wall related phenotypes, clear differences were observed between them and these differences depend on the drug used and the growth temperature. The lack of Mkk2 rendered cells more susceptible to Calcofluor White and Congo Red than the lack of Mkc1; interestingly, at higher temperatures (37 and 42°C) susceptibility to CFW was suppressed in both mutants. Such differences have been also reported in *S*. *cerevisiae* [54]. A possible explanation for this phenotype may reside in the role of Mkc1 as client protein to the CaHsp90 chaperone, which influences thermal adaptation in *C*. *albicans* by its concerted action with MAPK signalling pathways [30]. Depletion of Hsp90 has been shown to influence drug sensitivity and cell wall composition [55]. The existence of differences between *mkc1* and *mkk2* cells in cell wall related phenotypes is striking as Mkk2 is the only MAPKK activating Mkc1 under all conditions tested. While we do not have an definite explanation for this, it must be considered that MAPK deletion mutant phenotypes are not necessarily similar to those with inactive alleles [56], being possible that the Mkc1 protein still present in *mkk2* cells exerts a type of control in the CWI pathway, maybe preventing crosstalk with other pathways or occupying regulatory regions in the kinase complex. We also found differences with other cell wall stressors such as zymolyase, an enzymatic cocktail with a predominant β(1,3)-glucanase activity. *mkc1* mutants were sensitive while *mkk2* mutants behaved as a wt and deletion of *MKK2* restored wt phenotype in *mkc1* mutants. In *S*. *cerevisiae*, zymolyase-mediated cell wall damage activates both the HOG and PKC pathways, although it seems that the transcriptional response depends mainly on Slt2 [35]. We show here that *CRH11* induction does not take place in *mkc1* mutants at high temperatures but does in *mkk2*, confirming that the transcriptional response to environmental conditions in both mutants is different, which may contribute to explain this different behaviour. In fact, *CRH11* is an homologue of a glycosylphosphatidylinositol [GPI]-cell wall protein [42] that is involved in what has been called the “compensatory mechanism” [57] induced upon zymolyase treatment that regulates cell wall architecture. This different response may also be involved in a different behaviour in the interaction with host cells. Mkk2 does not seem to play a major role in invasion on solid surfaces in different media, a trait that has been shown to contribute to virulence [27, 28] (see [58] and [59] for recent reviews) and does not affect filamentation in the presence of serum. Survival of mice systemically infected is not altered in *mkk2* cells, and analyses *post mortem* do not reveal altered fungal burden of organs infected with *mkk2* cells. Similar results are observed in the *Galleria mellonella* insect model. Therefore, Mkk2, unlike Mkc1 [28], does not seem to contribute to virulence[28].

We have paid attention to the role of β(1,3)-glucan, the major component of the cell wall. This polymer is important for the maintenance of the cell wall, being normally hidden under a mannoprotein layer and only visible during division [43]. In *S*. *cerevisiae* it has been proposed that Slt2 plays a central role in a glucan masking network that occludes β(1,3)-glucan from immune cells [45]. Neither *mkc1* or *mkk2* had more exposed β(1,3)-glucan on their surfaces than wt, and the amount of exposed β(1,3)-glucan on the surface of an *hst7 mkk2* double mutant was not significantly different to that of an *hst7* single mutant Therefore, our data support the idea that in *C*. *albicans* this process seems to be mainly controlled by the Cek1-mediated pathway [46] and not by the CWI pathway (Fig 7). However, we also show that both Mkk2 and Mkc1 control the expression of the inducible essential β(1,3)-glucan synthase encoded by *CaGSC1* under defined conditions. Glucan synthesis is essential for fungal cells which, in *S*. *cerevisiae*, are accomplished via different genes: *ScFKS1*, *ScFKS2* (*GSC2*) and *ScFKS3*. *ScGSC2* is the orthologue of *CaGSC1*, whose expression is induced under starvation, during sporulation and in response to mating pheromones [60, 61]. In this organism, *ScFKS2* is a cell cycle regulated gene, controlled by the CWI pathway, and is activated in response to increases in temperature [62]. We show here that expression of *CaGSC1* is also induced upon a temperature increase in *C*. *albicans* and this is prevented in both *mkc1* and *mkk2* mutants, indicating similarities between both models. However, β(1,3)-glucan content does not seem to explain the differences in zymolyase sensitivity between both *mkk2* and *mkc1* mutants. It is possible that this may result from altered permeability in the cell wall arising from altered Cek1 expression as it has been shown that upstream elements of the Cek1 pathway severely influence the mannoprotein layer [63]. We show here that major differences between *mkc1* and *mkk2* mutants reside in Congo Red and Calcofluor White susceptibility, dyes that interfere with chitin assembly [64] that is partially regulated by the CWI pathway [17], coherent with this observation. However, in a semiquantitative analysis, we do not observe altered amounts of chitin in *mkk2* cell walls, as also occurs with *mkc1* mutants [25] suggesting that the defect may be either more related to specific linkages and/or organisation of the cell wall than alteration of absolute amounts.

Our results demonstrate that the impairment of both the CWI and *CEK1* pathways aggravate susceptibility to certain stresses. Double *hst7 mkk2* mutants are more susceptible to Calcofluor White and Congo Red as well as zymolyase (see Fig 5). Tunicamycin, an inhibitor of the first steps of N-glycosylation, triggered both Mkc1 and Cek1 phosphorylation. This phosphorylation was prevented in *mkc1* mutants, suggesting that Cek1 phosphorylation is partially dependent on Mkc1. Therefore, cell wall glycosylation defects in response to tunicamycin may trigger Mkc1 phosphorylation that, indirectly, may lead to Cek1 activation. Tunicamycin also induced Cek1 expression and this was independent of upstream elements of the pathway such as Msb2 and Sho1 [5]. Cek1 is not only regulated by phosphorylation but also at the transcriptional level and even via proteolytic degradation [39]. Cek1 becomes phosphorylated and its expression is induced when stationary growing cells at 24°C were refreshed in fresh pre-warmed medium and allowed to grow at 37°C. This induction is independent of elements of the Cek1-mediated pathway (R. Alonso-Monge, personal communication) but we show here that it depends on the CWI pathway. This reinforces the idea of the CWI pathway promoting Cek1 production as a cooperative mechanism that may be needed during certain conditions (growth resumption from stationary phase and temperature increase) where substantial cell wall remodelling may be needed (Fig 7). Both *mkc1* and *mkk2* mutants display similar sensitivity to tunicamycin, in agreement with the effect exerted on *CEK1* expression by both mutants. Therefore, Cek1 phosphorylation/expression may be relevant for tunicamycin resistance, as Msb2 and Sho1 (which mediate Cek1 phosphorylation) are sensitive to this inhibitor [4, 5]. While both the Cek1 and Mkc1-mediated pathways cooperate in cell wall biogenesis, share some triggering stimuli (zymolyase and tunicamycin) and phenotypes (susceptibility to Congo Red or tunicamycin), they still respond to separate stimuli and display separate phenotypes. Cek1 is activated in response to growth signals coming from release of *quorum sensing* molecules from stationary phase cells; this signalling mechanism is independent of Mkc1 and Mkk2. In parallel, Mkc1 is activated in response to oxidative stress [20], while this does not occur with Cek1. Therefore, the interaction between both pathways is dependent on the triggering stimulus which is closely linked to cell wall biogenesis (Fig 7). In conclusion, collectively our data support that both the CWI and Cek1-mediated pathways function during vegetative growth to promote a proper cell wall assembly and remodelling.

## Supporting Information

S1 Fig Cell cultures were diluted in YPD supplemented with tunicamycin at different concentrations at 24, 30, 37 and 42°C. Cultures were incubated overnight and growth was expressed as the percentage of growth in YPD supplemented with the cell wall interfering compound compared to growth in YPD alone. Mean values of 3 independent experiments (two samples per experiment) are represented with bars indicating the SD (standard deviation). Two-way ANOVA test was performed to assess differences (*, p<0.05; ****p<0.0001).
(TIF)

S2 Fig Overnight cells from cultures grown at 37°C were diluted and grown 18 hours at 24, 30, 37 and 42°C in the presence of different amounts of zymolyase starting at O.D._600_ = 0.025. Growth is depicted as the percentage of growth in YPD supplemented with the cell wall interfering compound compared to growth in YPD alone. Mean values are represented with bars indicating the SD (standard deviation) and two-way ANOVA test compare to the parental wild type strain was performed to evidence significant differences (**, p<0.01; ***, p<0.001 ****p<0.0001).
(TIF)

S3 Fig Detection of Cek1 in CWI mutants. Overnight cultures at 24°C of the indicated strains were shift to pre-warmed YPD medium at 24, 30, 37 and 42°C and incubated for 1 h. Extracts were obtained and Cek1 protein levels were detected using a polyclonal anti-Cek1 antibody.
(TIF)

S1 Table Relative expression of cell wall related genes. The levels of expression of each gene (*CEK1*, *GSC1* and *CRH11*) (compared to *ACT1*) at each temperature normalised to the value at 24°C for the wild type background are indicated for each of the mutant backgrounds analysed (wt, *mkc1*, *mkk2* and *mkc1 mkk2*). The relative abundance of *CEK1*, *GSC1* and *CRH11* mRNA at 24°C in wt cells was 1/14 ± 1.22, 1/35.7 ± 5.2 and 1/66.7± 6.21 compared to *ACT1* mRNA levels at the same temperature. n.d. Not determined. The statistical significance (p < 0.05) using the Holm-Sidak post hoc test after ANOVA is shown for each value for the comparison of wt with *mkc1* (a), wt with *mkk2* (b), wt with *mkc1 mkk2* (c) and wt with *cek1* (d).
(DOCX)
