# Supplementary figures and images for: The Mkk2 MAPKK Regulates Cell Wall Biogenesis in Cooperation with the Cek1-Pathway in Candida albicans
Source: PLoS One. 2015 Jul 21;10(7):e0133476. doi: 10.1371/journal.pone.0133476 (PMC4509911; doi:10.1371/journal.pone.0133476)

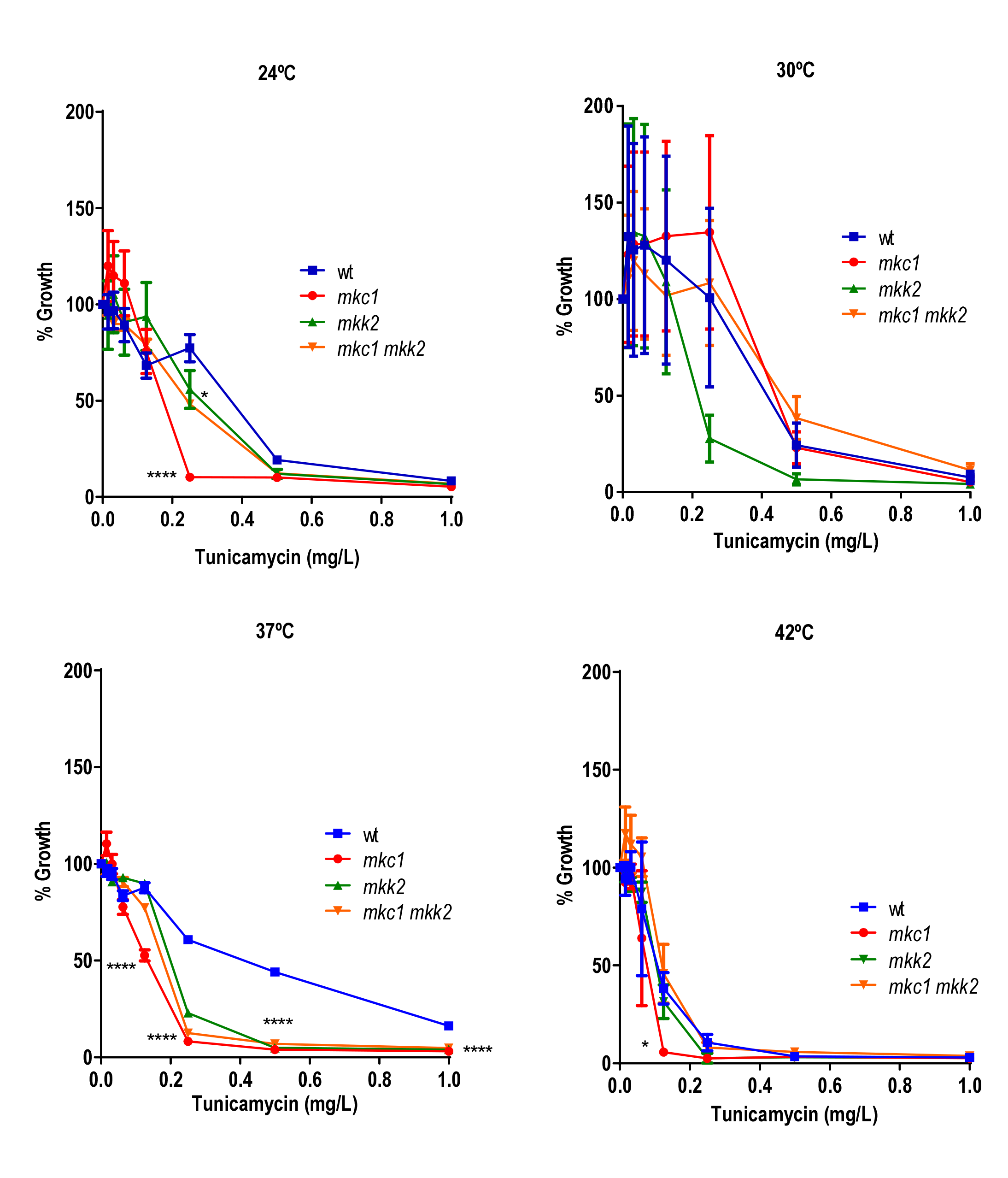

Supplement: S1 Fig — Cultures were incubated overnight and growth was expressed as the percentage of growth in YPD supplemented with the cell wall interfering compound compared to growth in YPD alone. Mean values of 3 independent experiments (two samples per experiment) are represented with bars indicating the SD (standard deviation). Two-way ANOVA test was performed to assess differences (*, p<0.05; ****p<0.0001). (TIF) [file pone.0133476.s001.tif]

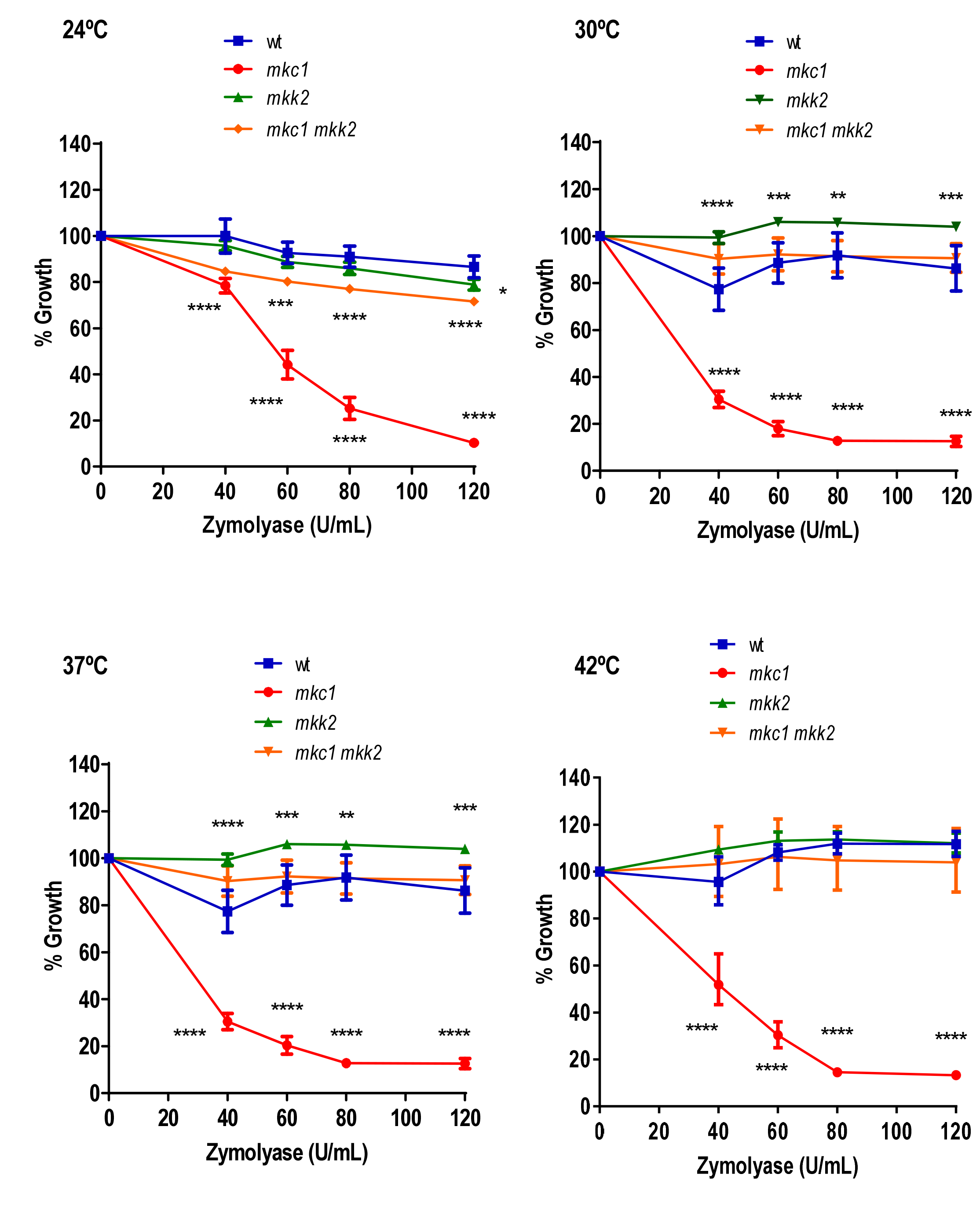

Supplement: S2 Fig — Growth is depicted as the percentage of growth in YPD supplemented with the cell wall interfering compound compared to growth in YPD alone. Mean values are represented with bars indicating the SD (standard deviation) and two-way ANOVA test compare to the parental wild type strain was performed to evidence significant differences (**, p<0.01; ***, p<0.001 ****p<0.0001). (TIF) [file pone.0133476.s002.tif]

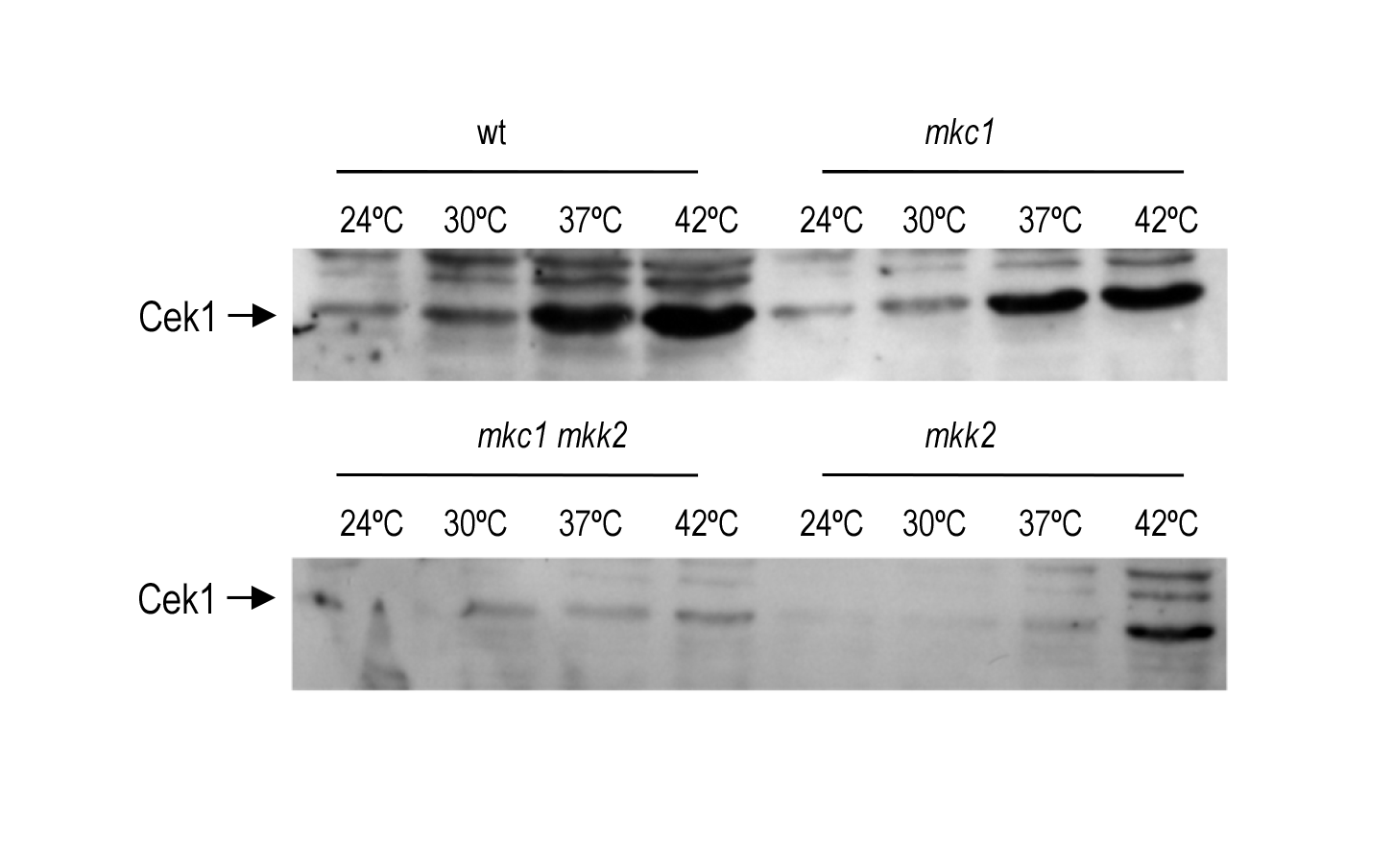

Supplement: S3 Fig — Overnight cultures at 24°C of the indicated strains were shift to pre-warmed YPD medium at 24, 30, 37 and 42°C and incubated for 1 h. Extracts were obtained and Cek1 protein levels were detected using a polyclonal anti-Cek1 antibody. (TIF) [file pone.0133476.s003.tif]
