# Supplementary material for: The Mkk2 MAPKK Regulates Cell Wall Biogenesis in Cooperation with the Cek1-Pathway in Candida albicans
Source: PLoS One. 2015 Jul 21;10(7):e0133476. doi: 10.1371/journal.pone.0133476 (PMC4509911; doi:10.1371/journal.pone.0133476)
Supplement: S1 Table — The levels of expression of each gene (CEK1, GSC1 and CRH11) (compared to ACT1) at each temperature normalised to the value at 24°C for the wild type background are indicated for each of the mutant backgrounds analysed (wt, mkc1, mkk2 and mkc1 mkk2). The relative abundance of CEK1, GSC1 and CRH11 mRNA at 24°C in wt cells was 1/14 ± 1.22, 1/35.7 ± 5.2 and 1/66.7± 6.21 compared to ACT1 mRNA levels at the same temperature. n.d. Not determined. The statistical significance (p < 0.05) using the Holm-Sidak post hoc test after ANOVA is shown for each value for the comparison of wt with mkc1 (a), wt with mkk2 (b), wt with mkc1 mkk2 (c) and wt with cek1 (d). (DOCX) [file pone.0133476.s004.docx]

**Supplementary Table 1. Relative expression of cell wall related genes**

|  | *CEK1* | | | | |  |
| --- | --- | --- | --- | --- | --- | --- |
|  | wt | *mkc1* | *mkk2* | *mkc1 mkk2* | *cek1* | **Significance** |
| 24 | 1.00 | 1.56 ± 0.05 | 0.79 ± 0.05 | 1.70 ± 0.05 | n.d. |  |
| 30 | 4.22 ± 1.01 | 1.17 ± 0.41 | 0.28 ± 0.15 | 2.18 ± 1.06 | n.d. |  |
| 37 | 14.49 ± 3.23 | 1.30 ± 0.498 | 1.28 ± 0.4 | 1.12 ± 0.21 | n.d. | a,b,c |
| 42 | 19.14 ± 2.17 | 19.13 ± 5.29 | 5.22 ± 0.17 | 5.20 ± 1.37 | n.d. | b,c |
|  | ***GSC1*** | | | | |  |
|  | wt | *mkc1* | *mkk2* | *mkc1 mkk2* | *cek1* |  |
| 24 | 1.00 | 1.55 ± 0.04 | 0.68 ± 0.16 | 1.35 ± 0.3 | 0.7 ± 0.08 |  |
| 30 | 7.11 ± 1.43 | 1.01 ± 0.36 | 0.50 ± 0.26 | 0.76 ± 0.23 | 2.8 ± 0.20 | a,b,c, |
| 37 | 21.88 ± 3.65 | 0.8 ± 0.12 | 1.05 ± 0.06 | 1.62 ± 0.06 | 5.3 ± 0.45 | a,b,c,d |
| 42 | 10.67 ± 1.32 | 2.72 ± 0.76 | 1.22 ± 0.9 | 0.9 ± 0.09 | 7.6 ± 0.62 | a,b,c |
|  | ***CRH11*** | | | | |  |
|  | wt | *mkc1* | *mkk2* | *mkc1 mkk2* | *cek1* |  |
| 24 | 1.00 | 1.55 ± 0.05 | 0.68 ± 0.16 | 1.32 ± 0.3 | 1.7 ± 0.08 |  |
| 30 | 18.04 ± 2.96 | 2.34 ± 0.39 | 1.12 ± 0.54 | 5.42 ± 2.11 | 1.9 ± 0.19 | a,d |
| 37 | 14.55 ± 2.20 | 10.92 ± 3.07 | 8.02 ± 3.17 | 13.23 ± 0.39 | 11.3 ± 2.64 | a,d |
| 42 | 4.74 ± 1.51 | 8.59 ± 0.31 | 24.27 ± 2.94 | 53.97 ± 4.11 | 33.3 ± 6.30 | b,c,d |
